# Sociobehavioral Risk Factors and Clinical Implications of Late Presentation Among People Living with HIV in the Brazilian Amazon Region

**DOI:** 10.1007/s10461-024-04437-5

**Published:** 2024-07-11

**Authors:** Leonn Mendes Soares Pereira, Eliane dos Santos França, Iran Barros Costa, Igor Tenório Lima, Amaury Bentes Cunha Freire, Francisco Lúzio de Paula Ramos, Talita Antonia Furtado Monteiro, Olinda Macedo, Rita Catarina Medeiros Sousa, Felipe Bonfim Freitas, Igor Brasil Costa, Antonio Carlos Rosário Vallinoto

**Affiliations:** 1 Virology Laboratory, Institute of Biological Sciences, Federal University of Pará, Belém, Pará Brazil; 2 Epstein‒Barr Virus Laboratory, Virology Unit, Evandro Chagas Institute, Ananindeua, Pará Brazil; 3 Epidemiology and Surveillance Service, Evandro Chagas Institute, Ananindeua, Pará Brazil; 4 Retrovirus Laboratory, Virology Unit, Evandro Chagas Institute, Ananindeua, Pará Brazil; 5 School of Medicine, Federal University of Pará, Belém, Pará Brazil; 6 Graduate Program in Virology, Evandro Chagas Institute, Ananindeua, Pará Brazil

**Keywords:** HIV, Late presentation, Risk factors, Amazon, Brazil

## Abstract

This study aimed to analyze the prevalence, sociobehavioral factors and clinical-laboratory consequences of late presentation among people living with HIV (PLHIV) in the Brazilian Amazon region. In total, 402 HIV + individuals treated at reference units in Belém city (Pará, Brazil) between 2018 and 2019 were evaluated. Late presentation was defined as a first-collection LTCD4^+^ count below 350 cells/µL. Sociodemographic, behavioral and clinical data were obtained from questionnaires or medical records. Th1, Th2 and Th17 cytokine profiles were evaluated by flow cytometry. Longitudinal data on viral load, T lymphocytes, and antiretroviral therapy administration were obtained from control and logistic databases. Approximately 52.73% of the participants were late presenters and sought medical care 7–12 + months after their primary HIV diagnosis. Sociobehavioral factors associated with late presentation included illicit drug use for more than 5 years, polyamory, no alcohol consumption, homosexuality, and sexual inactiveness after HIV diagnosis. Clinically, late presentation was associated with coinfection rate; polysymptomatology; high IFN-ɣ, IL-6 and IL-10 levels; nonresponse to antiretroviral therapy; and virological failure- and tuberculosis coinfection-motivated changes to therapy. In summary, the prevalence of late presentation in Pará in the Brazilian Amazon region is high. Delays in seeking specialized care after a primary HIV diagnosis cause medium/long-term changes in the life expectancy and health of PLHIV.

## Introduction

The first scientific reports that introduced the topic of late presentation conceptualized it as a late manifestation of symptoms indicative of AIDS, according to the definitions of the time, in both asymptomatic and symptomatic individuals. These manifestations were not specific, although some immunological indicators, such as a CD4^+^ T lymphocyte (LTCD4^+^) count below the normal range, were already observed in “late patients” [1, 2].

Only in mid-1992 was a behavioral connotation attributed to the concept of “late presentation”, in an Australian study in which the development of AIDS-defining symptoms was associated with a delay in specialized care, which, at the time, was due to a lack of knowledge about HIV infection, concerns about confidentiality, and the belief that the patient’s sexual exposures had been relatively “safe” in relation to the risk of HIV infection [3]. In a subsequent study performed in a developed country, although late presentation was not associated with sociodemographic or ethnic characteristics or even the LTCD4^+^ count, its impact was shown in terms of the severity of the immunodeficiency presented [4].

With the resulting reports, late presentation has begun to encompass behavioral and temporal perspectives with a very specific reflection on the LTCD4^+^ count, which is now a clinical biomarker of the condition. In these terms, late presenters are considered all people living with HIV (PLHIV) who, due to the delay in seeking specialized care, have an LTCD4^+^ count below expectations or present to the clinic with an AIDS-defining event. However, although most studies adopt a cutoff of < 350 cells/µL, there is still some discrepancy regarding the cutoff point for the LTCD4^+^ T-cell count. In more specific cases, an advanced condition is indicated by a compromised immunological status (LTCD4^+^ <200 cells/µL), the presence of multiple opportunistic infections and general mental and functional health problems [5–12].

In developed countries, the most frequent sociodemographic profile among late presenters included being elderly, male, heterosexual, from an underdeveloped country and, less frequently, a drug user [8, 9, 13–16]. In underdeveloped countries, late presenters are male, homosexual, have a low educational level, live far from urban centers and still suffer from fear of stigma related to the condition [17–22].

In Brazil, many late presenters seek care when the infection is at a more advanced stage [23]. In the central-southern region of the country, the findings indicate a late presentation frequency ranging from of 11.2–56.7% of the total number of PLHIV sampled, and the main factors associated with the condition were age over 35 years, non-white ethnicity, level of social vulnerability (education and income) and, in some cases, refusal to accept specific care and treatments [24–27]. In the North and Northeast, the average number of cases of late presentation varies by 60%, and the strongest risk factors include homosexual orientation, age over 30 years, use of illicit drugs and low education [28, 29]; there is a warning about increasing the adjusted prevalence estimates for pregnant women [30] and people who work under hostile conditions in unhealthy environments [31].

According to estimates, circumstances are worsening; approximately 46% of PLHIV, especially those in underdeveloped countries, are unaware of their serological status [32]. The costs of this lack of knowledge include an increase in individual morbidity and mortality, the risk of transmission to sexual partners, and the decreased effectiveness of subsequent steps in the infection treatment cascade [33]. Although the literature contains many studies on risk factors related to late presentation, there is still debate about the extent to which these data allow the characterization of late presentation for care and treatment purposes, as well as the real correlation of these aspects with the immunological status of patients [34].

This has become more common in the Brazilian Amazon, where notification of infection remains low [35], and we did not identify studies that address the epidemiological aspects of late presentation and their respective impacts on the clinical management of PLHIV in the region. Only one guiding study carried out with PLHIV admitted to a hospital in the Amazon region highlighted that in patients with an LTCD4^+^ count > 350 cells/µL, there was a high prevalence of opportunistic coinfections, such as pulmonary tuberculosis, neurotoxoplasmosis and candidiasis oropharyngeal–esophageal infections, with a mortality rate of 20.5% [36].

Based on the lack of reports on the epidemiological status of late presentation and its consequences at the level of the Brazilian Amazon region, we aimed to evaluate the prevalence of late presenters of HIV-1 infection treated in reference outpatient units in the capital of the State of Pará, to identify sociobehavioral risk factors related to the condition; and to evaluate the clinical consequences of late presentation regarding symptoms, history of coinfections and the dynamics of maintaining antiretroviral therapy. In a challenging space regarding the planning and supervision of public policies such as the Brazilian Amazon region, we work with the hypothesis of the high prevalence of late presenters as a direct reflection of the population’s social vulnerability and with subsequent repercussions regarding the management and care of the quality of life of PLHIV.

In this way, we seek to understand the regional factors associated with late presentation as a fundamental basis for the development of counseling and care strategies for PLHIV in the context of the Brazilian Amazon.

## Methods

### Sampling and Ethical Aspects

This study was carried out through partnerships signed among the Virology Laboratory of the Federal University of Pará (LABVIR-UFPA), the Evandro Chagas Institute (IEC) and the Center for Health Care in Acquired Infectious Diseases (CASA DIA), with the weekly selection of samples from prescreened PLHIV or those with suspected infections residing in the state of Pará, Brazil, from January 2018 to January 2019.

In compliance with resolutions 466/2012 and 347/05 of the National Health Council, this project was submitted for ethical consideration and approved by the IEC Human Research Ethics Committee (CEP) (Protocol: 3.121.265; CAAE: 73927717.3. 0000.0019), and all methods were carried out in accordance with the principles of the Declaration of Helsinki, following the recommendations provided by the guidelines for reporting observational studies. All participants were informed about the research objectives and signed informed consent forms.

Contact and assessment of patients by the project team occurred once. During this time, biological samples and sociodemographic and behavioral data were collected from each patient, and medical records were analyzed to obtain data relating to symptoms and reports of coinfections at that time.

Upon consent from patients and referral units, we obtained access to prospective data regarding T lymphocyte measurement, viral load quantification, the type of therapeutic regimen prescribed and the maintenance or exchange of therapeutic regimens, when applicable, for each patient to evaluate the effects of late presentation on the evolution of HIV infection over the two-year survey period.

The following individuals were eligible to participate in this study: (i) individuals of either sex; (ii) individuals infected with HIV; (iii) individuals who were not undergoing antiretroviral treatment (ART) at the time of biological material collection; and (iv) individuals aged 18 years or older. The following individuals were excluded from this study: (i) participants who did not have continuous data on the monitoring platforms and (ii) participants who did not answer all the questions proposed to obtain data. Ultimately, 402 PLHIV participated in this study.

We defined late presenters as PLHIV with an LTCD4^+^ T-cell count > 350 cells/µL measured at initial patient intake.

### Sociodemographic, Behavioral and Clinical Data

All sociodemographic and behavioral data were obtained through the application of a questionnaire with objective multiple-choice questions to obtain information from each participant on the following: the probable date of diagnosis of HIV infection; sex; age; schooling; family income; the use of legal and illicit drugs and, in the latter case, the duration of their use; sexual orientation; whether they were sexually active; whether they maintained monogamous relationships; whether they had had sexual contact with sex workers; whether they used condoms during sexual acts; the most recurrent symptomatologic complaints; and whether they had comorbidities and/or coinfections.

Data on each participant’s symptoms, comorbidities and coinfections were obtained from his or her medical records, and the results of anamnesis and laboratory tests requested by trained professionals from the reference centers were also collected.

Regarding symptoms, the patient was classified as asymptomatic when there were no symptoms. Oligosymptomatic was defined as the presence of up to 3 symptoms, and polysymptomatic was defined as the presence of 4 or more symptoms.

### Screening, Quantification and Grouping

Suspected HIV infection was confirmed by qualitative detection of the p24 antigen and anti-HIV-1 and anti-HIV-2 IgG antibodies by enzyme immunoassay (Murex AG/AB Combination Diasorin, UK); serological confirmation was performed using a DPP HIV-1/2 rapid immunoblot kit (Bio-Manguinhos, FIOCRUZ) following the manufacturer’s recommendations. The CASA DIA samples did not require complementary diagnostic tests since the institution has its own screening panel, which was used for the enrolled patients.

The plasma HIV viral load was quantified by real-time PCR using the Abbott mSample Preparation System RNA Extraction Kit and the Abbott Real-Time HIV-1 Amplification Matrix (ABBOTT, Chicago, Illinois, USA) following the manufacturer’s recommendations.

The quantification of CD4^+^ T (CD45^high^CD3^+^CD4^+^CD8^−^) and CD8^+^ T (CD45^high^CD3^+^CD4^−^CD8^+^) lymphocytes was performed by immunophenotyping and flow cytometry using BD FACSCalibur-4-color equipment and the FACSCountTM reagents and TriTEST™/TruCount monitoring kits (BD Biosciences, San Jose, CA, USA), following the manufacturer’s recommendations.

The plasma concentrations of the cytokines IL-17 A, IFN-ɣ, TNF, IL-10, IL-6, IL-4 and IL-2 were determined by cytometric bead array (CBA) using BD FACSCanto™ II equipment and the BD™ CBA Human Th1/Th2/Th17 Cytokine Kit (BD Biosciences, San Jose, CA, USA).

### Monitoring and Therapeutic Response Profiles

After the first collection, lymphocyte and viral load quantification data were obtained from the Laboratory Test Control System of the National Network for CD4^+^/CD8^+^ T Lymphocyte Counting and HIV Viral Load (SISCEL) through interval consultations every six months; the quantification methods used for each period were the same as those previously described. Data on the management and distribution of antiretrovirals were obtained from the Medication Logistic Control System (SICLOM), and data were collected every six months, during which the initial therapeutic schemes, the changes in approach and the corresponding reasons were determined. Data collection took place over a period of twenty-four months (2 years) from the date of the first collection, at which point none of the PLHIV had undergone therapy.

After one year of highly active ART (HAART), we stratified PLHIV according to the profile of the immune response to treatment into groups of nonresponders (LTCD4^+^ <500 cells/µL; varied viral load) and responders (LTCD4^+^ ≥500 cells/µL; varied viral load), according to recent publications [37].

### Statistical Analysis

The comparison of sociodemographic, behavioral, clinical and therapeutic data between late presenters and non-late presenters was carried out using the chi-square (x^2^) and G tests, according to recommendations for performing each analysis [38].

Sociobehavioral risk factors were selected by univariate and multivariate logistic regression analyses.

We calculated Spearman’s coefficient to evaluate the degree of correlation between cytokine concentrations and lymphocyte counts, such as the plasma viral loads of late presenters and non-late presenters. We opted for the nonparametric test due to the degree of normality of the variables in question, which was estimated using the Lilliefors test. Quantitative data were compared between groups in 2-by-2 analyses using the Mann‒Whitney test (U) and between 3 or more groups through analysis of variance using the Kruskal‒Wallis test (H).

For the statistical analyses, we adopted an α of 5%, considering a probability of significance (*p*) less than or equal to 0.05 as a rejection criterion for null hypotheses. All calculations were performed using GraphPad Prism 8.4.3 (San Diego, CA, USA), BioEstat 5.3 software [38] and the R platform [39]. We constructed radar graphs using Excel (2010) to show the frequency of types of coinfections, types of symptoms and reasons for changing therapeutic regimens for late and non-late presenters.

## Results

### Prevalence and Sociobehavioral Risk Factors Associated with Late Presentation

**Fig. 1:**
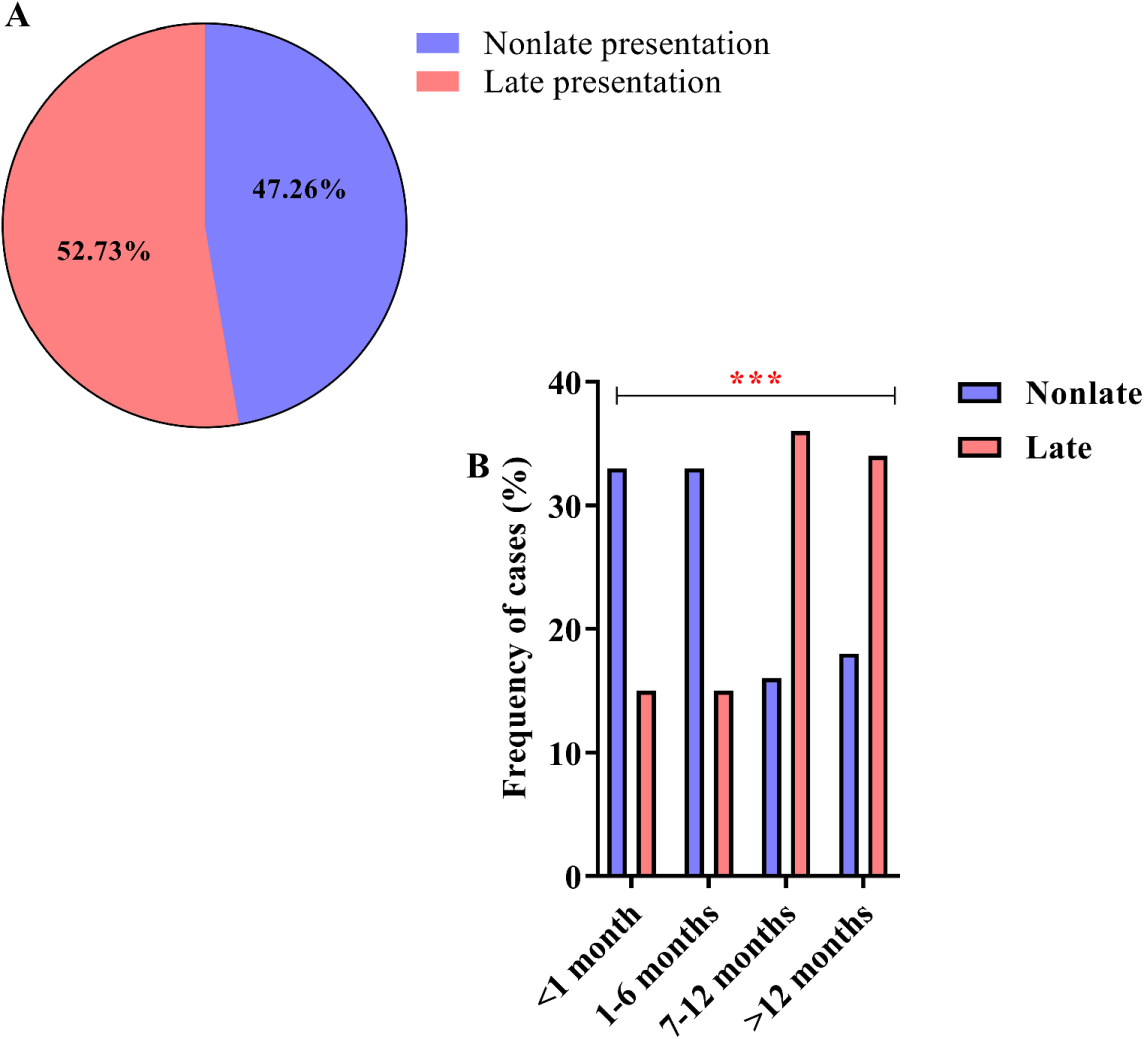
Proportion of late presenters. (**A**) “Parts of a whole” graph showing the frequencies of late presenters and non-late presenters. (**B**) Column graph showing the percentage of time spent seeking specialized care at referral units after the primary diagnosis of HIV (***: *p* < 0.0001)

Among the 402 PLHIV analyzed, 190 (47.26%) were non-late presenters, while 212 (52.73%) were late presenters (Fig. 1A). We observed a significant association between late presentation and the time spent searching for specialized care in referral units after the primary diagnosis of HIV; among individuals considered to be late presenters, approximately 36% (76 of 212) of PLHIV experienced a delay in care that lasted from 7 to 12 months, and 34% (72 of 212) experienced a delay in care that lasted more than 12 months (G-test = 53.97, *p* < 0.0001) (Fig. 1B).

The late presenters had the following characteristics: (i) they were residents of the metropolitan region of Belém, and most of them were residents of the capital itself; (ii) they were male; (iii) they were between 18 and 28 years old; (vi) they had completed primary education; (v) they had a family income between 1 and 3 minimum wages; (vi) they reported alcoholism; and (vii) the majority were not users of illicit drugs. However, among those with a history of illicit drug use, the duration of continuous use was more than 5 years.

Among the sociobehavioral factors with adjustments in the multiple logistic regression, we observed that individuals with alcoholism had a lower chance of late presentation than did those without alcoholism (Z value= -2.4004, OR = 0.36, 95% CI = 0.16–0.83, *p* = 0.015). Conversely, individuals who used of illicit drugs use for more than 5 years had a higher chance of having a late presentation than did nondrug users (Z value = 1.9646, OR = 1.82, 95% CI = 1.00-3.31, *p* = 0.042). Both homosexuals (Z value= -3.8029, OR = 0.24, 95% CI = 0.12–0.46, *p* < 0.0001) and bisexuals (Z value= -4.3023, OR = 0.39, 95% CI = 0.24–0.64, *p* = 0.0001) had lower chances of late presentation than did heterosexuals. Individuals with an active sexual life had lower chances of late presentation than did those without an active sexual life (Z value= -2.0368, OR = 0.61, 95% CI = 0.38–0.98, *p* = 0.035). Individuals without a steady sexual partner had a higher chance of late presentation than did those with a steady sexual partner (Z value = 3.7606, OR = 2.55, 95% CI = 1.57–4.16, *p* = 0.0001) (Table 1).

**Table 1:** Description of social and behavioral aspects among PLHIV with non-late and late presentation

Presentation	Non-late presentation	Late presentation	Univariate logistic regression			Multivariate logistic regression		
Factors	190 (47.26)	212 (52.73)	Z value	OR (95% CI)	*p* values	Z value	OR (95% CI)	*p* values
Sex								
Women	39 (20.52)	42 (19.81)		**Ref**				
Men	151 (79.47)	170 (80.19)	0.1813	1.05 (0.64–1.70)	0.856			
**Age**								
18–28	125 (65.79)	92 (43.40)		**Ref**				
29–39	40 (21.05)	63 (29.72)	3.1196	2.14 (1.33–3.47)	0.002			
40–50	18 (09.47)	41 (19.34)	3.5997	3.10 (1.66–5.75)	0.003			
51 or more	7 (03.68)	16 (07.55)	2.3983	3.11 (1.23–7.88)	0.017			
**Education**								
Illiterate or literate	23 (12.11)	24 (11.32)	0.5315	3.04 (0.82–11.4)	0.097			
Elementary School 1	34 (17.89)	36 (16.98)	1.1970	2.30 (0.59–9.02)	0.231			
Elementary School 2	86 (45.26)	82 (38.68)	1.0275	1.94 (0.55–6.86)	0.304			
High School	39 (20.53)	66 (31.13)	1.6584	1.42 (0.39–5.17)	0.595			
University education	8 (04.21)	4 (01.89)		**Ref**				
**Family income**								
Not determined	10 (05.26)	18 (08.49)	0.0242	1.70 (0.59–4.90)	0.326			
(< 1) salary	32 (16.84)	84 (39.62)	2.0249	2.63 (1.15–5.98)	0.022			
(1–3) salary	133 (70.00)	95 (44.81)	2.0228	0.72 (0.34–1.54)	0.040			
(> 3) salary	15 (07.89)	15 (07.08)		**Ref**				
**Smoking**								
No	79 (41.58)	100 (47.17)		**Ref**				
Yes	111 (58.42)	112 (52.83)	-1.1228	0.80 (0.54–1.18)	0.262			
**Alcoholism**								
No	10 (05.26)	29 (13.68)		**Ref**			**Ref**	
Yes	180 (94.74)	183 (86.32)	-2.8151	0.33 (0.15–0.71)	0.005	-2.4004	0.36 (0.16–0.83)	0.015
**Illicit drugs**								
No	135 (71.05)	133 (62.74)		**Ref**				
Yes	55 (28.95)	79 (37.26)	1.8754	1.50 (0.98–2.28)	0.061			
**Time of use**								
No use	135 (71.05)	133 (62.74)		**Ref**			**Ref**	
< 5 years	32 (16.84)	24 (11.32)	-1.0520	0.73 (0.41–1.31)	0.293	-1.1508	0.65 (0.39–1.42)	0.273
> 5 years	23 (12.11)	55 (25.94)	3.2744	2.47 (1.43–4.24)	0.001	1.9646	1.82 (1.00-3.31)	0.042
**Sexual orientation**								
Heterosexual	52 (27.37)	109 (51.42)		**Ref**			**Ref**	
Homosexual	100 (52.63)	79 (37.26)	-4.2254	0.38 (0.25–0.60)	< 0.0001	-3.8029	0.24 (0.12–0.46)	< 0.0001
Bisexual	38 (20.00)	24 (11.32)	-3.8326	0.30 (0.17–0.56)	0.0001	-4.3023	0.39 (0.24–0.64)	0.0001
**Active sex life**								
No	55 (28.95)	100 (47.17)		**Ref**			**Ref**	
Yes	135 (71.05)	112 (52.83)	-3.7397	0.45 (0.30–0.69)	0.0002	-2.0368	0.61 (0.38–0.98)	0.035
**Steady sexual partner**								
No	93 (48.95)	127 (59.91)	4.7251	2.72 (1.80–4.13)	< 0.0001	3.7606	2.55 (1.57–4.16)	0.0001
Yes	97 (51.05)	85 (40.09)		**Ref**			**Ref**	
**Sexual relationships with sex workers**								
No	150 (78.95)	153 (72.17)		**Ref**				
Yes	40 (21.05)	59 (27.83)	1.4619	1.73 (0.70–3.48)	0.653			
**Condom use after diagnosis**								
Never	22 (11.57)	30 (14.15)	1.4338	1.46 (0.73–2.89)	0.281			
Always	168 (88.42)	182 (85.85)		**Ref**				

**Ref**: Reference. **IQR**: Interquartile range

## Association of Late Presentation with Coinfections and Symptomatology

**Fig. 2:**
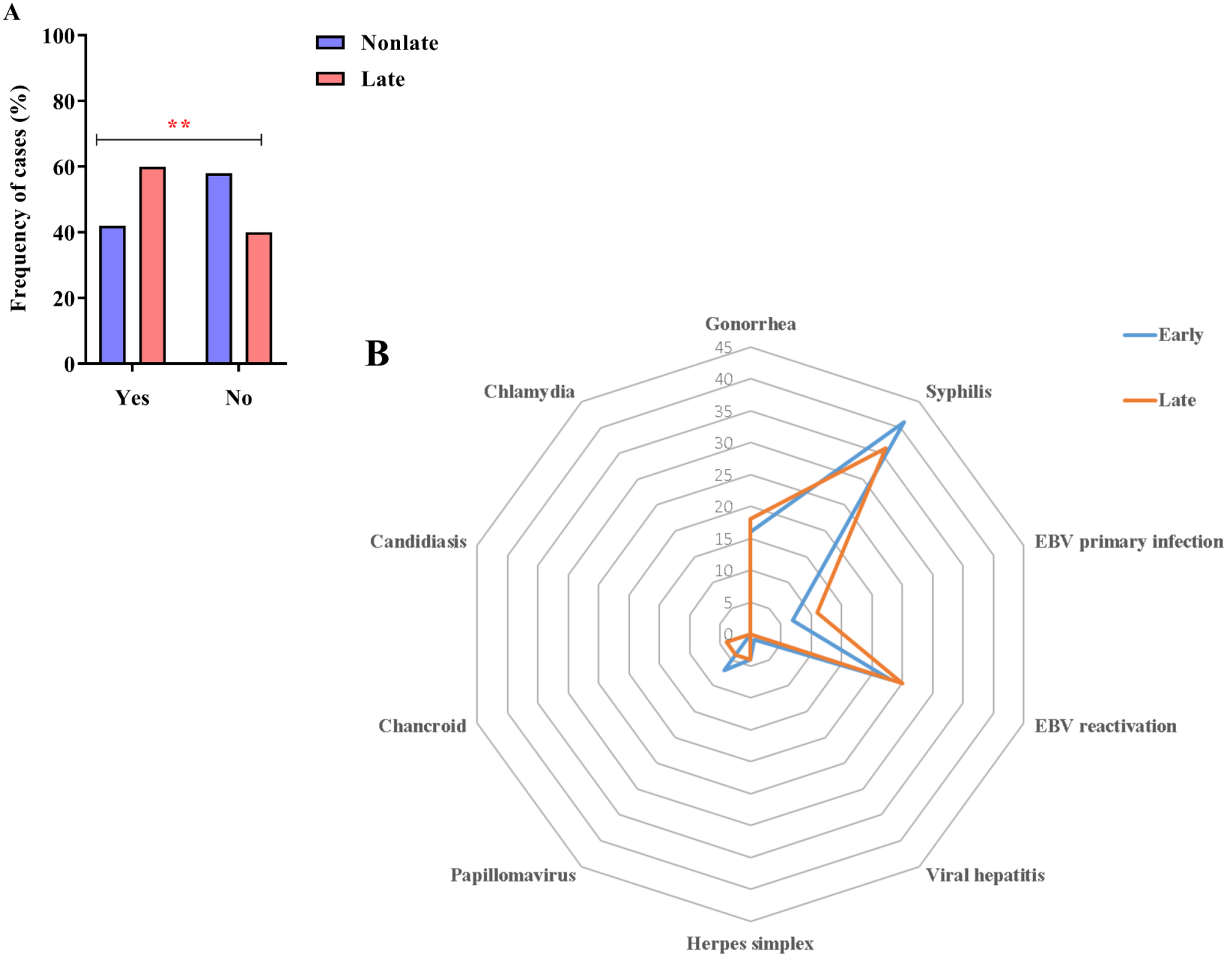
Coinfection. (**A**) Column chart showing the high frequency of coinfection cases among late presenters (**: 0.05 < *p* < 0.01). (**B**) Radar graph showing that syphilis and EBV reactivation were the most frequent coinfections in the PLHIV studied

A history of coinfections was prevalent in 59.91% (127 of 212) of late presenters, whereas 42.11% (80 of 190) of non-late presenters, had such a history (χ^2^ = 12.008, *p* = 0.001) (Fig. 2A). The most frequent coinfections were syphilis (36–41%) and Epstein–Barr virus (EBV) reactivation (23–25%) in both groups; however, the frequency of chancroid among late presenters was 3% (6 of 212), which was higher than expected (G-test = 22.9526, *p* = 0.014) (Fig. 2B).

**Fig. 3:**
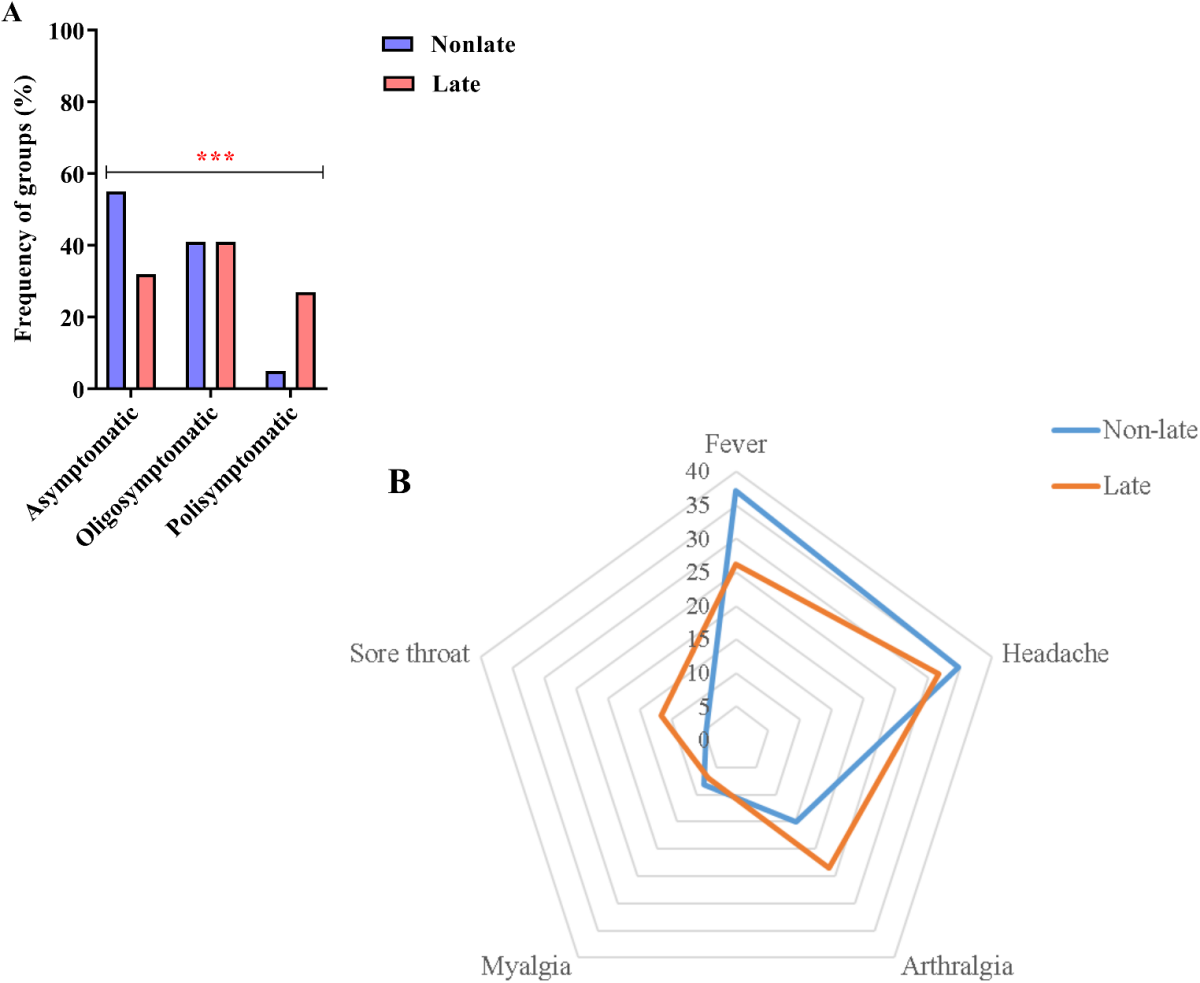
Symptomatology. **(A)** Graph in columns showing the frequency of symptomatic groups among late and non-late presenters (***: *p* < 0.0001). **(B)** Radar graph showing the frequency of symptoms manifested by late and non-late presenters

Regarding symptomatology, asymptomatic cases were more common among non-late presenters (54.74%; 104 out of 190) than among late presenters (31.6%; 67 out of 212); oligosymptomatic cases were more frequent among late presenters (41.04%; 87 out of 212) than among non-late presenters (40.53%; 77 out of 190); and polysymptomatic cases were more prevalent among late presenters (27.36%; 58 out of 212) than among non-late presenters (4.74%; 9 out of 190) (χ^2^ = 43.377, *p* < 0.0001) (Fig. 3A). According to the symptom profile of late presenters, headache was the most frequent complaint (31.72%; 38 of 145 symptomatic), and myalgia was the least frequent complaint (6.89%; 10 of 145 symptomatic). In non-late presenters, the most common complaint was fever (37.21%; 32 of 86 symptomatic), and sore throat was the least frequent complaint (4.65%; 4 of 86 symptomatic). However, when the symptom profiles of the two groups were compared, there were no significant differences (G-test = 7.5883, *p* = 0.1234) (Fig. 3B).

### High Levels of IFN-γ, IL-6 and IL-10 in Late Presenters

**Fig. 4:**
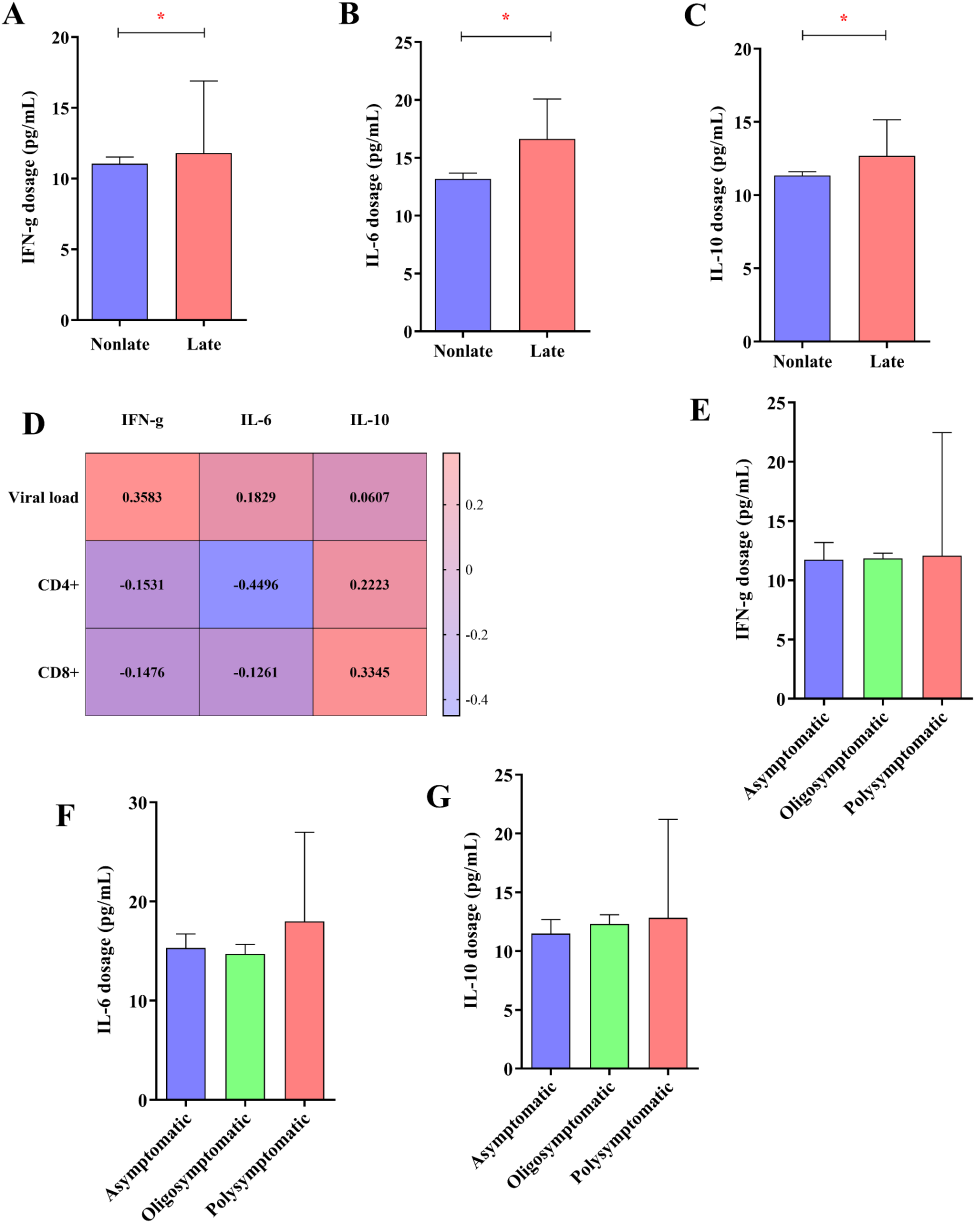
Cytokine dosage. Column graphs showing elevated levels of the cytokines IFN-ɣ (**A**), IL-6 (**B**) and IL-10 (**C**) in late and non-late presenters. (**D**) Heatmap showing the Spearman coefficients between the concentration of IFN-ɣ, IL-6 and IL-10, the HIV viral load, and the concentrations of CD4^+^ and CD8^+^ T lymphocytes (*: *p* < 0.005). Column graphs showing the concentrations of IFN-ɣ (**E**), IL-6 (**F**) and IL-10 (**G**) according to the grouping of symptoms of late presenters

The serum levels of the cytokine IFN-ɣ were higher in late presenters (median = 11.80, IQR = 6.82) than in non-late presenters (median = 11.06, IQR = 3.67) (U = 2854, *p* = 0.039) (Fig. 4A). The serum levels of the cytokine IL-6 were higher in late presenters (median = 16.62; IQR = 8.07) than in non-late presenters (median = 13.16, IQR = 5.79) (U = 2735, *p* = 0.019) (Fig. 4B). The serum levels of the cytokine IL-10 were higher in late presenters (median = 12.68, IQR = 4.80) than in non-late presenters (median = 11.35, IQR = 3.39) (U = 3029, *p* = 0.048) (Fig. 4C).

Figure 4D shows the correlation between cytokine levels and several markers of the pathology of HIV infection, in which the IFN-ɣ level was correlated with viral load (*r* = 0.358; *p* = 0.045); however, it was not associated with CD4^+^ T lymphocyte count (*r*= -0.153; *p* = 0.138) or CD8^+^ T lymphocyte count (*r*= -0.148; *p* = 0.153). The IL-6 level was correlated with viral load (*r* = 0.183; *p* = 0.046) and CD4^+^ T lymphocyte count (*r*= -0.450; *p* = 0.015); however, it was not associated with CD8^+^ T lymphocytes (*r*= -0.126; *p* = 0.223). The IL-10 level was correlated with only the CD8^+^ T lymphocyte count (*r*= -0.335; *p* = 0.043).

We did not observe any associations between the serum levels of IFN-ɣ (H = 0.2054, *p* = 0.902) (Fig. 4E), IL-6 (H = 3.041, *p* = 0.219) (Fig. 4F) or IL-10 (H = 1.232, *p* = 0.540) (Fig. 4G) and the presence of symptoms in individuals in the group of late presenters.

### Maintenance of Concerning Viral Loads and LTCD4^+^ Counts in Late Presenters EvenAfter ART

**Fig. 5:**
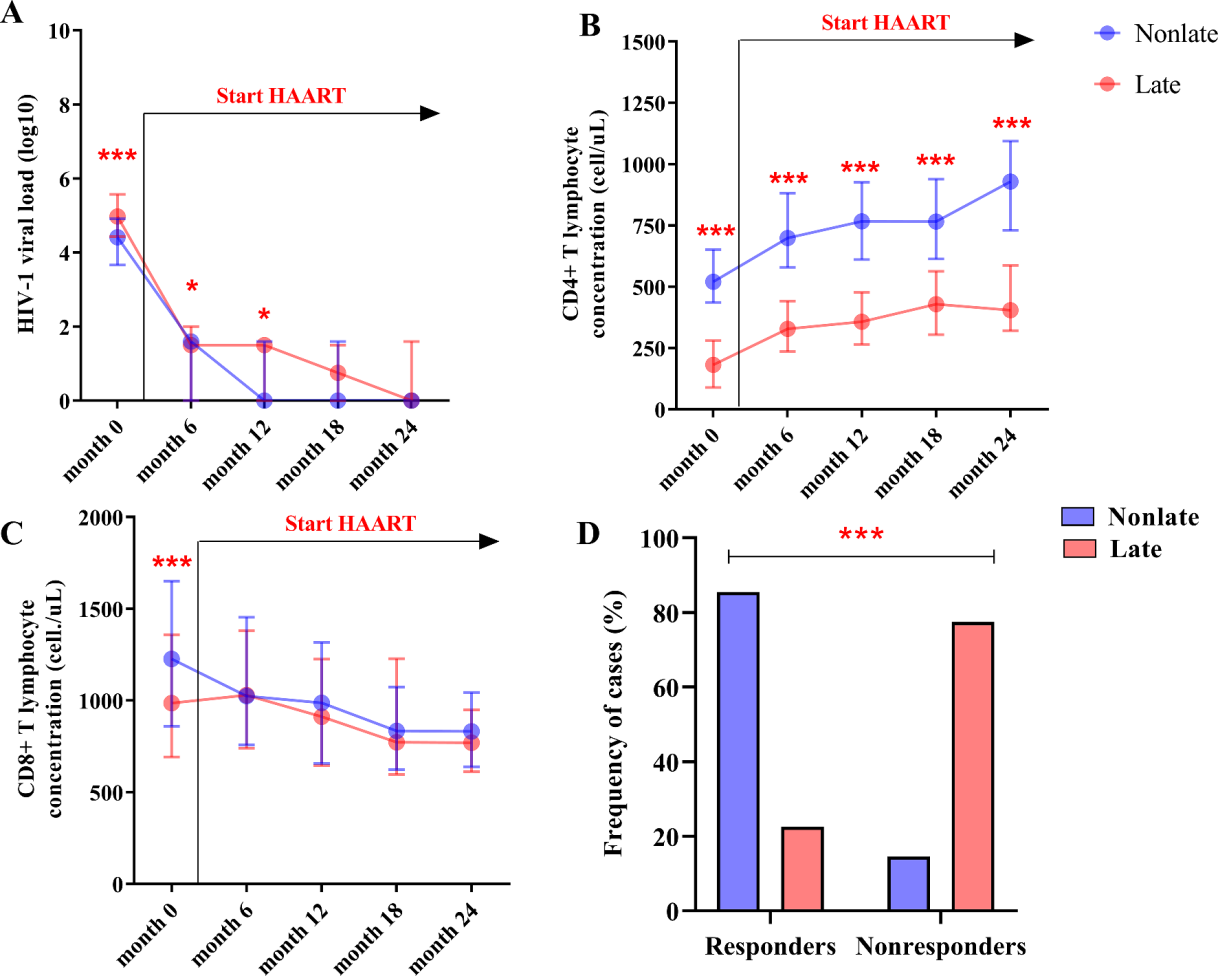
Changes in the viral load and T lymphocyte concentration. Diagram of extremes and quartiles showing the evolution of viral load (**A**), CD4^+^ T lymphocyte concentration (**B**) and CD8^+^ T lymphocyte concentration (**C**) over the course of the 24 months of evaluation (*: *p* < 0.05), (**: 0.05 < *p* < 0.01), (***: *p* < 0.0001). (**D**) Column plot showing the high frequency of therapeutic nonresponders among late presenters (***: *p* < 0.0001)

Late presenters had a high HIV viral load (log_10_) at month 0 before ART (median = 4.97; IQR = 1.14). Six months after starting ART, there was a reduction in viral load (log_10_)in both groups; however, viral load in late presenters remained significantly high (median = 1.50; IQR = 2.00). The viral load (log_10_) became undetectable in most non-late presenters by the 12th month of continued ART use (median = 0; IQR = 1.60); however, in most late presenters, the viral load (log_10_) became undetectable only after twenty-four months of ART (median = 0; IQR = 1.60). After 12 months of ART, the viral load (log_10_) of late presenters was still detectable (median = 1.50; IQR = 1.53) (Table 2; Fig. 5A).

**Table 2:** Comparison of viral load data, CD4^+^ T and CD8^+^ T lymphocyte counts between late and non-late presenters during the two years of sample collection

Variables	Non-late presentation		Late presentation		U	*p* values
	Median	IQR	Median	IQR		
Viral load (log_10_)						
Month 0	4.41	1.25	4.97	1.14	11778.50	< 0.0001
Month 6	1.60	1.60	1.50	2.00	13564.50	0.027
Month 12	0	1.60	1.50	1.53	10941.50	0.041
Month 18	0	1.60	0.75	1.50	6628.00	0.081
Month 24	0	0	0	1.60	1103.00	0.459
**CD4** ^**+**^ **T lymphocyte count**						
Month 0	520	216	182	192	4698.50	< 0.0001
Month 6	699	303	329	206	2768.50	< 0.0001
Month 12	767	315	357	212	2034.50	< 0.0001
Month 18	766	325	429	258	1535.50	< 0.0001
Month 24	929	363	404	266	1256.00	< 0.0001
**CD8** ^**+**^ **T lymphocyte count**						
Month 0	1226	791	986	667	15395.50	< 0.0001
Month 6	1024	697	1030	642	14493.50	0.590
Month 12	987	660	912	581	8168.00	0.290
Month 18	834	451	772	631	4871.00	0.716
Month 24	832	405	770	337	1049.50	0.296

**U**: Manny-Whitney test

The CD4^+^ T lymphocyte concentration was low in late presenters at month 0, as expected (median = 182.0; IQR = 192). Notably, we observed that the recovery of CD4^+^ T lymphocytes remained at suboptimal proportions in late presenters compared to non-late presenters throughout the 24 months of ART use. The discrepancy in values between the two groups was highly significant (*p* < 0.0001) in all observation periods (Table 2; Fig. 5B). This was reflected in the percentage of therapeutic nonresponders, which was higher in the group of late presenters (77.48%, 181 of 212) than in the group of non-late presenters (22.52%, 28 of 190) (χ^2^: 200.318, *p* < 0.0001) (Fig. 5D).

We monitored changes in the concentration of CD8 + T lymphocytes just before the start of ART and found that the changes in CD8 + T lymphocytes in non-late presenters were greater than those in late presenters (median = 1226; IQR = 791) (Table 2; Fig. 5C).

### Late Presentation Associated with Therapy Exchange

**Fig. 6:**
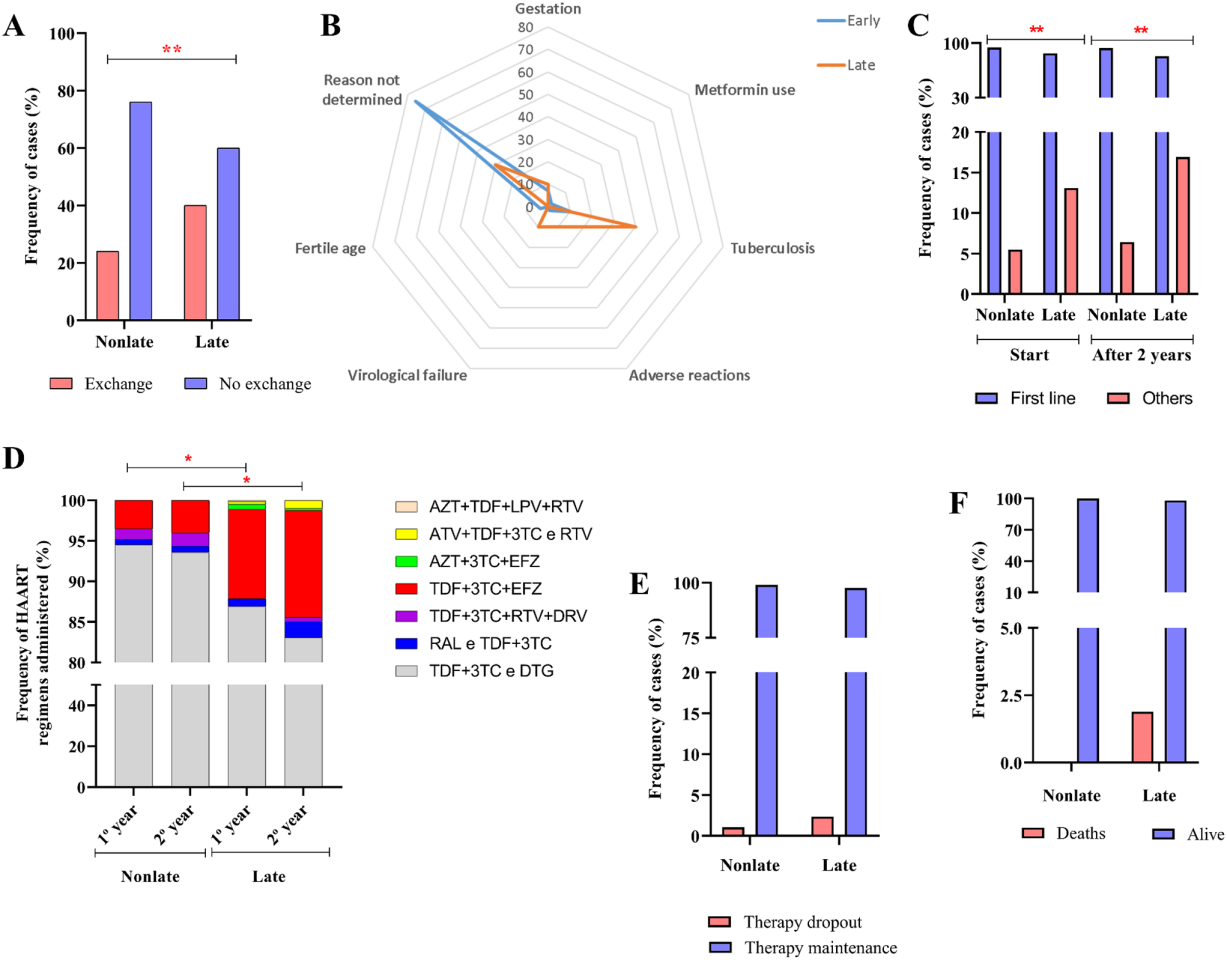
ART dispensing and maintenance. (**A**) Column graph showing the high rate of ART switching among late presenters. (**B**) Radar graph showing that tuberculosis infection and virological failure were the reasons for ART switching more frequently than expected in late presenters. (**C**) Column graph showing the high frequency of choosing alternative ART regimens among late presenters both at the start of treatment and after 2 years of continuous use. (**D**) Column graph showing the diversity of ART regimens administered over the 24 months of follow-up. (**E**) Column graph showing the ART maintenance and abandonment rates. (**F**) Column graph showing the rate of death during the follow-up period (*: *p* < 0.05), (**: 0.05 < *p* < 0.01)

The total rate of ART switching was greater in the late-presentation group (40%; 85 of 212) than in the non-late-presentation group (23.68%; 45 of 190) (χ^2^ = 12.332, *p* = 0.001) (Fig. 6A). Tuberculosis (40%; 32 of 212) was the most common reason for switching among late presenters; however, the frequency of therapeutic changes due to virological failure was higher than expected in the same group (10%; 9 of 212). Reasons for switching the ART were not reported for most non-late presenters (75.44%; 33 of 190) (G-test = 37.4456, *p* < 0.0001) (Fig. 6B).

We analyzed the choice of first-line therapy regimens comprising tenofovir, lamivudine, and dolutegravir (TDF + 3TC and DTG) [40] and alternative regimens among non-late and late presenters (Fig. 6C). Most PLHIV in both groups were treated with the first-line regimen; however, we observed that the percentage of patients who discontinued alternative regimens was higher among late introducers. Over the course of one year of therapy, approximately 13.10% (28 of 212) of late presenters had already started treatment with alternative regimens, while among non-late presenters, the percentage of patients who received alternative regimens increased to approximately 5.49% (10 of 190) (χ^2^ = 7.388, *p* = 0.007). Over two years of therapy, there was a slight increase to 16.92% (36 of 212) in the use of alternative regimens among late presenters, which was significant compared to the frequency of use among non-late presenters 6.42% (12 of 190) (χ^2^ = 10.840, *p* = 0.001).

After one year of therapy, the distribution of treatment regimens differed between the PLHIV groups (G-test = 13.8211, *p* = 0.032), in which approximately 95% (180 of 190) of non-late presenters used the TDF + 3TC and DTG regimens; in this same group, the frequency of administration of the tenofovir + lamivudine, ritonavir and darunavir (TDF + 3TC + RTV + DRV) regimen was higher than expected (1%, 2 of 190); and in late presenters, the tenofovir + lamivudine + efavirenz (TDF + 3TC + EFZ) regimen was administered to 11% of patients (23 of 212) (Fig. 6D). With two years of therapy, the differences in schedule distribution remained significant (G-test = 16.5692, *p* = 0.011); the TDF + 3TC and DTG regimens were administered to approximately 94% (178 of 190) of non-late presenters, whereas the TDF + 3TC + EFZ regimen was administered to approximately 13% (28 of 212) of late presenters (Fig. 6D).

The therapeutic dropout rates were low and nonsignificant, but the values were higher among late presenters (2.36%; 5 of 212) (G-test = 1.0380, *p* = 0.5334) (Fig. 6E). Similarly, the death rate was low in both groups, but the value was higher value among late presenters (1.89%; 4 of 212) (G-test = 2.5865, *p* = 0.5119) (Fig. 6F).

## Discussion

In the present study, 52.73% of the participants were late presenters and sought specialized care between 7 and more than 12 months after the primary diagnosis of the infection. This frequency was higher than expected compared to that in industrialized countries [41]; however, it is within the estimate observed in underdeveloped countries [42].

It is alarming that this substantial delay in seeking treatment is undoubtedly associated with the risk of progression of infection and transmission, such as the increased risk of viral resistance to therapy and the acceleration of immune failure [43]. On the one hand, this failure may be a consequence of structured stigma, especially in low- and middle-income countries [44]; on the other hand, it is due to the negligence of individuals diagnosed with the infection who start care after a prolonged period, usually after the onset of symptoms [45]. We suggest that negligence was one of the main reasons for the scenario presented if we account for the similarity of the sociodemographic profile observed in the present study, at some points, to the findings of Krawczyk et al. [46], who also concluded that negligence in patients was the factor most related to late presentation. Interestingly, some participants commented that the search for specialized care occurred only due to the influence of third parties or the onset of symptoms (data not shown).

Among the factors adjusted for late presentation highlighted in the present study, long-term illicit drug use and sexual promiscuity without a steady partner are widely associated with the challenges of managing and counseling PLHIV in different populations [46, 47]. However, in terms of late presentation, the literature shows an apparent dichotomous trend in sociobehavioral profiles between studies in developing countries and those in more developed countries. In the latter, late presentation appears to be associated with advanced age and sexual behavior [48, 49], while in developing countries, including Brazil, or in underdeveloped countries, late presentation is associated with ethnic factors and poor social indicators [17–22]. We also did not rule out regional influences on the findings since these are epidemiological factors regularly associated with HIV-1 infection in the Brazilian Amazon region [50].

The predominance of reports of limited alcohol consumption and sexual abstinence were curious findings among late presenters. Steward and colleagues reported that the diagnosis of infection can play an important role in reducing transmission risk behaviors over time, but they also highlighted the need for interventions to improve and sustain these changes [51]. The authors reported behaviors related to failures in sexual prevention, while in the present study, even with the reduction in excessive alcohol consumption and sexual abstinence, late presenters who consumed illicit drugs for more than 5 years predominated. Furthermore, although some changes in attitudes were favorable in reducing collective risks, the delay in seeking specific care continued to be crucial for the poor prognosis of the infection in late presentation cases.

Drastic pathological features related to late presentation were observed in the present study. Regarding the rate of coinfections, approximately 60% of late presenters had coinfections before starting ART; curiously, the frequency of chancroid cases was higher than expected in this group in relation to the population as a whole. A simple search of public databases revealed that between 1992 and 2022, there was a decrease in publications on the prevalence of chancroid cases among PLHIV, which appears to predominate in populations with minimal access and/or precarious social structures [52, 53]. However, in Brazil, complementary data are scarce or neglected, as they associate coinfection with a very low amount of CD4^+^ T lymphocytes [54], which is a preponderant characteristic of the late presenters in the present study.

Thus, we call attention to the emergence of sexually transmitted infections (STIs), which are uncommon, especially in late-presenting PLHIV. The high frequency of cases of syphilis and EBV reactivation, which are coinfections related to the poor prognosis of HIV-1 infection [55, 56], confirms this discussion. In particular, approximately 12–14% of the participants still did not use condoms even after HIV diagnosis.

Regarding morbidity, approximately 27.36% of the late presenters were polysymptomatic, with headache being the most common complaint in the group analysis. There are in-depth studies on the pathophysiology of headache in patients with HIV, which is a multifactorial condition that can occur at any time during the infection and can be directly linked to HIV or due to opportunistic diseases and inflammatory immune restoration syndrome [57].

The interesting aspect of these studies is that the authors associate these manifestations with no use or discontinued use of the therapy, disfavoring the course of the good prognosis expected with the administration of ART. As an indication, we show a reciprocal symptomatological profile in late presenters, with correlated symptoms that together disqualify individual well-being. Interestingly, participants who complained of fever did not claim sore throat, arthralgia or headache, a trend also observed among non-late presenters.

Immunological instability is expected in PLHIV who do not adhere to ART [58]. We observed that in late presenters, IFN-ɣ, IL-6 and IL-10 levels were high and correlated with other markers of infection.

The ambiguous role of IFN-ɣ, which can both favor the maintenance of the restrictive immune response to infection through the activation of signaling pathways still under study [59] and favor the replication of HIV when in tune with the β-catenin signaling pathway [60], is discussed. In the present study, the cytokine dosage was directly proportional to the viral load, although it was negatively related to the concentration of T lymphocytes, indicating an unfortunate effect of IFN-ɣ superregulation on late presenters, which may have contributed to the high viral load and the low concentration of CD4^+^ T lymphocytes observed in this group.

In addition, increased IL-6 levels were significantly associated with low CD4^+^ T lymphocyte counts, which in fact has already been observed in baseline studies and is associated with immune dysfunction [61]. Likewise, the increase in IL-10 associated with a high concentration of CD8^+^ T lymphocytes may be due to the emergence of subsets of these cells that coordinate an adaptive regulatory response to immune activation during chronic infection [62].

This immunological imbalance was reflected longitudinally, as both the viral load and the concentration of CD4^+^ T lymphocytes in late presenters remained at suboptimal proportions even after the initiation of ART, accounting for 77.48% of therapeutic nonresponders after one year of therapy. In the same group, the rate of switching from ART to nonprimary schemes based on EFZ, as a substitute for DTG, was frequent and independent of the analysis time.

It was expected that the concentration of CD4 + T lymphocytes would increase after the initiation of ART since the general recovery of cell frequency is observed even in late presenters [63, 64]. However, other studies have shown that even in late presenters starting therapy, immunological recovery remains impaired both in the short [65] and long term [66]. This may be associated with the notable increase in the frequency of antiretroviral resistance mutations in late presenters, mainly in nucleoside transcriptase inhibitors [67, 68], which compromises patients’ expectations of recovering their prognosis.

Our results reaffirm the very serious therapeutic failure to which late presenters are subjected, as demonstrated by Truong et al. (2006) [69] when reporting 17 cases of failure and resistance to ART, of which 12 were patients had long-term infection and a late diagnosis.

We observed that the main causes of changing therapy in late presenters were virological failure and tuberculosis. These data are alarming since it has already been shown that virological failure is entirely related to ART switching in late presenters [69], with adherence to treatment being a recognized predictor of therapeutic success [70]. In Brazil, this is particularly worrying if we consider the prevalence of antiretroviral resistance to nucleoside reverse transcriptase inhibitors, nonnucleoside reverse transcriptase inhibitors and protease inhibitors, mainly in the Brazilian Amazon region [71, 72], because these inhibitors make up the main regimens administered to the PLHIV population [73].

In Brazil, recent data indicate that the occurrence of tuberculosis continues to be associated with high mortality rates in PLHIV in both outpatient surveys [74] and intensive care units [75]. Although it is suggested that mortality is associated with tuberculosis acquired prior to treatment [76], other results show that treatment failure is related to the acquisition of tuberculosis and, consequently, the progression of HIV over at least 2 years of intensive therapy [77], as also observed in the present study.

Switching from DTG to EFZ was a plausible measure for obtaining successful virological results, especially in the context of coinfection with *M. tuberculosis* [40, 78]. However, the approach presented a clear failure in the attempt at immunological reconstitution of late presenters, which may be a consequence of the reduction in thymic function and the death of CD4^+^ T lymphocytes by pyroptosis [79], as well as the overcommitment of innate lymphoid cells due to late initiation of ART [80]. These points serve as a perspective for future studies on late presentation.

The main limitation of this study was the use of only prospective data regarding the provision of HAART and the quantification of viral load and T lymphocytes. A more comprehensive follow-up that accounts for the behavioral and clinical aspects of late presenters over time can be used to evaluate the evolution of this group more precisely.

## Conclusion

In conclusion, we found that the prevalence of late presentation was high in a cohort of PLHIV living in the State of Pará (Brazilian Amazon). Our results serve as a warning regarding how a delay in seeking specialized care after the primary diagnosis of HIV can result in medium- and long-term changes in the life expectancy and health of PLHIV. Therefore, we recommend more specific attention to the early treatment of PLHIV, considering their sociobehavioral characteristics.

In a future study, it will be important to identify the reasons underlying the delay in seeking specialized care among late presenters. With this knowledge as a supplement to the findings of the present study, we hope to provide a scientific basis for the proposal of public policies that decrease and mitigate the frequency of late presentation in the Brazilian Amazon region.

## Data Availability

The data generated in the present study are not available in public databases for the appreciation of readers and journal editing teams, being under the management of the co-senior authors and the first author. Their availability requires a request to the corresponding author.
